# Level of neo-epitope predecessor and mutation type determine T cell activation of MHC binding peptides

**DOI:** 10.1186/s40425-019-0595-z

**Published:** 2019-05-22

**Authors:** Hanan Besser, Sharon Yunger, Efrat Merhavi-Shoham, Cyrille J. Cohen, Yoram Louzoun

**Affiliations:** 1 The Leslie and Susan Gonda Multidisciplinary Brain Research Center, Bar-Ilan University, 5290002 Ramat Gan, Israel; 2 Department of Mathematics, Bar-Ilan University, 5290002 Ramat Gan, Israel; 3 Ella Lemelbaum Institute for Immuno Oncology, Sheba Medical Center, Ramat Gan, Israel; 4 Laboratory of Tumor Immunology and Immunotherapy, Goodman Faculty of Life Sciences, Bar-Ilan University, Ramat Gan, Israel

**Keywords:** T cell activation, Machine Learning, Neoantigen, MHC-binding peptides

## Abstract

**Background:**

Targeting epitopes derived from neo-antigens (or “neo-epitopes”) represents a promising immunotherapy approach with limited off-target effects. However, most peptides predicted using MHC binding prediction algorithms do not induce a CD8 + T cell response, and there is a crucial need to refine the predictions to readily identify the best antigens that could mediate T-cell responses. Such a response requires a high enough number of epitopes bound to the target MHC. This number is correlated with both the peptide-MHC binding affinity and the number of peptides reaching the ER. Beyond this, the response may be affected by the properties of the neo-epitope mutated residues.

**Methods:**

Herein, we analyzed several experimental datasets from cancer patients to elaborate better predictive algorithms for T-cell reactivity to neo-epitopes.

**Results:**

Indeed, potent classifiers for epitopes derived from neo-antigens in melanoma and other tumors can be developed based on biochemical properties of the mutated residue, the antigen expression level and the peptide processing stage.

Among MHC binding peptides, the present classifiers can remove half of the peptides falsely predicted to activate T cells while maintaining the absolute majority of reactive peptides.

**Conclusions:**

The classifier properties further highlight the contribution of the quantity of peptides reaching the ER and the mutation type to CD8 + T cell responses. These classifiers were then validated on neo-antigens obtained from other datasets, confirming the validity of our prediction.

Algorithm Availability: http://peptibase.cs.biu.ac.il/Tcell_predictor/ or by request from the authors as a standalone code.

## Introduction

CD8 T cell activation by either exogenous or endogenous epitopes is induced by binding of the T cell receptor to epitopes presented on host MHC class I proteins. Such peptides are usually the product of cytosolic protein or DRIP eventually digested by the proteasome [1]. The cellular TAP apparatus transfers cleaved peptides from the cytosol to the ER, where they can bind the MHC protein (for TAP dependent MHC binding). The probability of each of the above steps is often determined by the peptide linear sequence. This strongly simplifies the prediction of these stages by computational tools. We and others have developed multiple such tools for MHC binding, TAP binding and proteasomal cleavage [2, 3]. However, recent evidence suggests that most peptides predicted or measured to bind the MHC do not necessarily induce a T cell response when tested in vitro with a given patient’s T cells. Understanding the underlying reasons for this lack of optimal predictions represents an important challenge in T cell based anti-tumor treatments.

Progress in the understanding of immune components and their function has led to the implementation of successful immunotherapeutic approaches [4] based on checkpoint inhibitors or the adoptive transfer of tumor–specific T-cells. These strategies were shown to mediate regression of large tumor masses and remission in terminally-ill patients with different malignancies [5]. T-cells targeting cancer cells can recognize antigens which can be classified into two broad categories. The first class are non-mutated proteins or “tumor associated antigens” whose restricted tissue expression pattern probably allows for an immune response in patients [6]. The second class, on which we focus herein, are antigens derived from mutated proteins that could be recognized as foreign, commonly termed “neo-antigens”. These antigens have been shown to be relevant in efficient CPI (Check-point inhibitors) treatment and in T-cell based therapies [4]. Neoantigens can be used to identify tumor specific T-cells [7] or generate vaccines [8]. While advances in genomic sequencing have enabled a better characterization of DNA mutations and these antigens, an in silico approach to predict potent T-cell epitopes is lacking [9]. Experimental verifications are therefore needed [10], and these have revealed that most MHC-binding peptides are not recognized by T-cells. This could be the result of “holes” in the T cell repertoire or from properties of the peptides or the antigen from which they originated (e.g., protein expression level or biochemical properties of the peptides). Mass spectrometry can be applied for direct identification of epitopes [11], though the yield is often low and necessitates large amounts of tumor cells which are not always available.

Thus, we sought here to devise a novel algorithm based on experimentally acquired data to predict which MHC binding peptide would also induce a T cell response.

**Fig. 1:**
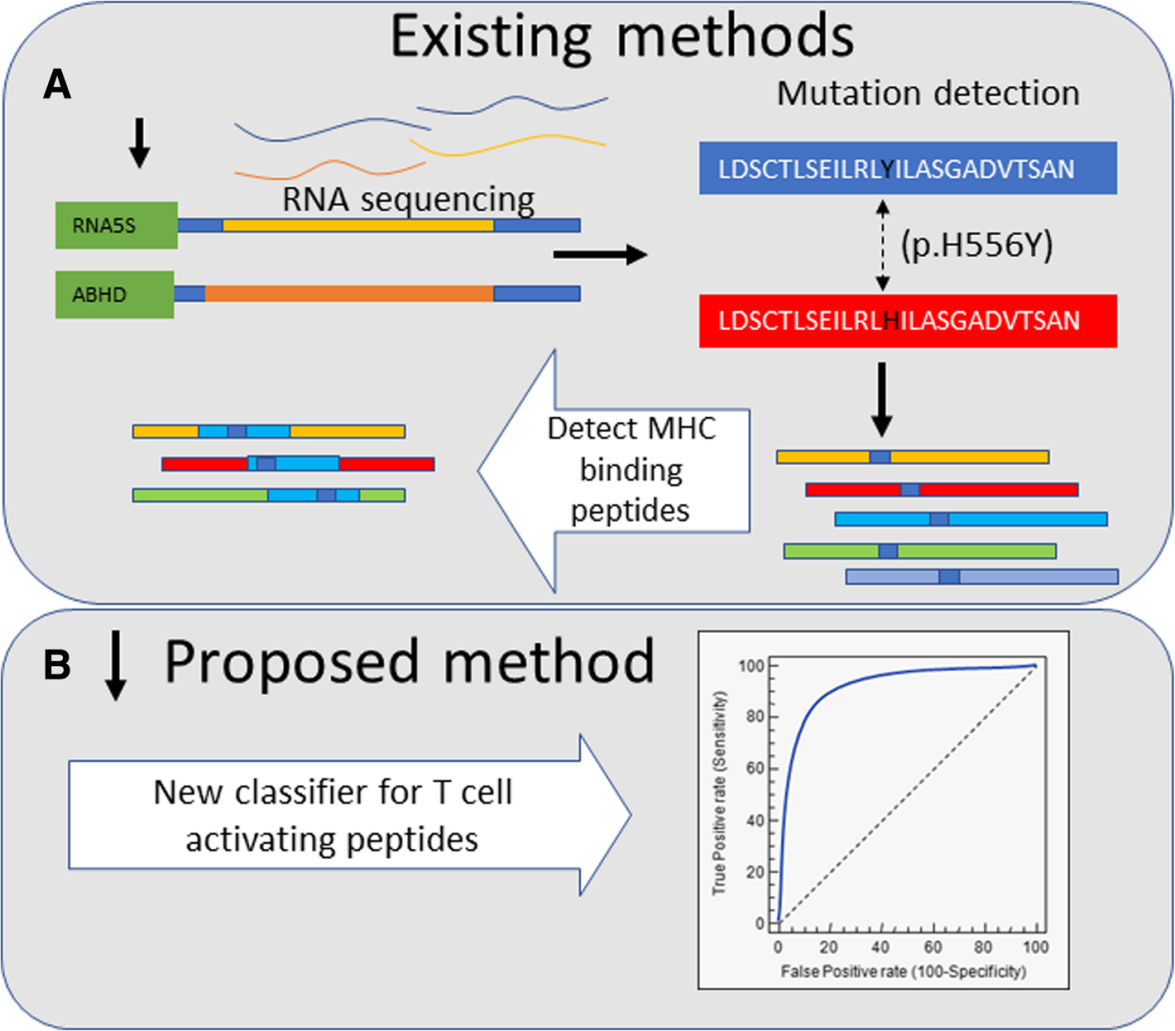
Existing methods and proposed new classifier (**a**) Current approaches for neo-antigen detection involve three main stages: RNA sequencing, detection of mutations in tumor cells and the computation of MHC binding peptides in such mutated regions. We propose a new stage (**b**) the detection among the MHC binding peptides of those that manage to induce a T cell response

The most natural and expected prediction filter for neo-antigens is their binding to MHC, and multiple tools were developed to predict this binding [2, 3] with high precision and fidelity. Indeed, an accuracy of over 95% of predicting whether a peptide would induce a response can be obtained using only MHC binding [12, 13]. In parallel, methods were developed to study the immunogenicity of presented peptides. In contrast with epitope presentation, epitope immunogenicity is a function of the TCR recognition of MHC-I/epitope complex, and does not rely only on the peptide binding per se. Recent results show that large and aromatic R-chains in certain positions in the peptides can affect T cell activation [14]. Hydrophobicity has also been shown to induce immunogenicity, while polarity seems negatively correlated to immunogenicity. Multiple predictors were developed for the immunogenicity of peptides [15]. However, those are here shown to be of limited use in neoantigens, and there is currently a need for tools to predict the activation of T cells by MHC binding peptides. The novel algorithm presented here is intended to be used after the majority of peptides not binding MHC have been discarded using MHC binding predictions [16, 17] to improve on those (Fig. 1).

Several groups have studied specific class I epitopes in neo-antigens and have also shown that epitopes from low expression proteins or from poorly preprocessed proteins do not induce a response [18, 19]. Other studies focused on the difference of the activation of the immune system between the W.T. and the mutant peptide by sequence property (with known algorithms), and involved other measures of the mutant peptide. Such methods report an Area Under Curve (AUC) of 0.63 [20].

Beyond MHC binding, the next candidate for affecting T cell response is the number of peptides reaching the Endoplasmic Reticulum (ER). This number determines the number of candidate MHC binding peptides, and in viral responses, we have shown it to be tightly related to the escape mutations [21]. We thus test the effect of the amount of such predecessors on the T cell response. Moreover, the response may be affected by the properties of the mutations producing the neo-antigen. Such properties were thus also added to the analysis.

## Results

### Correlation between neo-antigen features and T cell activation

In order to study the factors affecting T cell activation by presented peptides and to develop a classifier for such peptides, we analyzed the response of T-cell cultures derived from eight metastatic melanoma patients and published positive epitopes against sets of predicted-HLA binders as previously described [7] (further denoted the Me. Dataset). We also studied existing large scale datasets of T cell activating peptides, such as the Tantigen [22] dataset (further denoted the M.T. database). Finally, we tested three new melanoma patients studied in different experimental protocols to ascertain that our methods maintain their validity when applied to data produced by different experimental methodologies (Table 1).

**Table 1:** Summary of the datasets used

Source	HLA	Total no. of predicted samples	Confirmed Positive samples	Confirmed Negative samples
Melanoma (Me.)	A*02:01	485	35	450
Melanoma Patient 1	A*02 B*18, B*35 C*07, C*05	187	7	180
Melanoma Patient 2	A*11, A*23 B*14, B*41	56	3	53
Melanoma Patient 3	A*02, A*24 B*15, B*38	68	3	65
Tantigen [44]	mix		24	0

For each dataset the name of the dataset, the number of positives and negatives epitopes in the data, and the HLA composition of the data are presented. The Melanoma patients were used for validation of the model and the results

Multiple tools were previously developed to predict T cell activation by MHC binding peptides [2, 3], the main one being the IEDB Class I Immunogenicity tool [23]. However, when we tested the quality of this predictor in the specific context of neo-antigen datasets, the results obtained were an AUC of 0.51 (i.e., random).

To develop an accurate predictor for T-cell activation, we computed seven measures per peptide, including the expression level of the specific gene in tumor tissues in this specific type of tumor (taken from gene expression measurements), four measures representing differences between the mutant candidate epitope and the non-mutated sequence, including size, hydrophobicity, charge, and polarity. The parameters of size, hydrophobicity, and charge represent the absolute value of the change. The last two measures are the candidate epitope cleavage and tap binding probabilities (Table 2).

**Table 2:** First column is the score name, second column is the description of the score

Feature name	Description	Notes
Expression level	The average expression level by cell line in melanoma tissue	http://www.ebi.ac.uk/
Size difference	The absolute difference in size between the W.T. amino acid and the mutant	By Dalton units
Hydrophobicity	The absolute difference in hydrophobicity index between the W.T. amino acid and the mutant	Kyte J, Doolittle RF
Charge difference	The absolute difference in charge between the W.T. amino acid and the mutant	Values at ph = 7.4
Polar change	Categorical variable for the polarity change between the W.T. amino acid the mutant	Values at ph = 7.4
Cleavage score	Estimated cleavage probability of a full peptide.	Vider et al.
Tap score	Estimated TAP binding energy	Peters et al.

Third column is a description of the score and the reference for the score

**Fig. 2:**
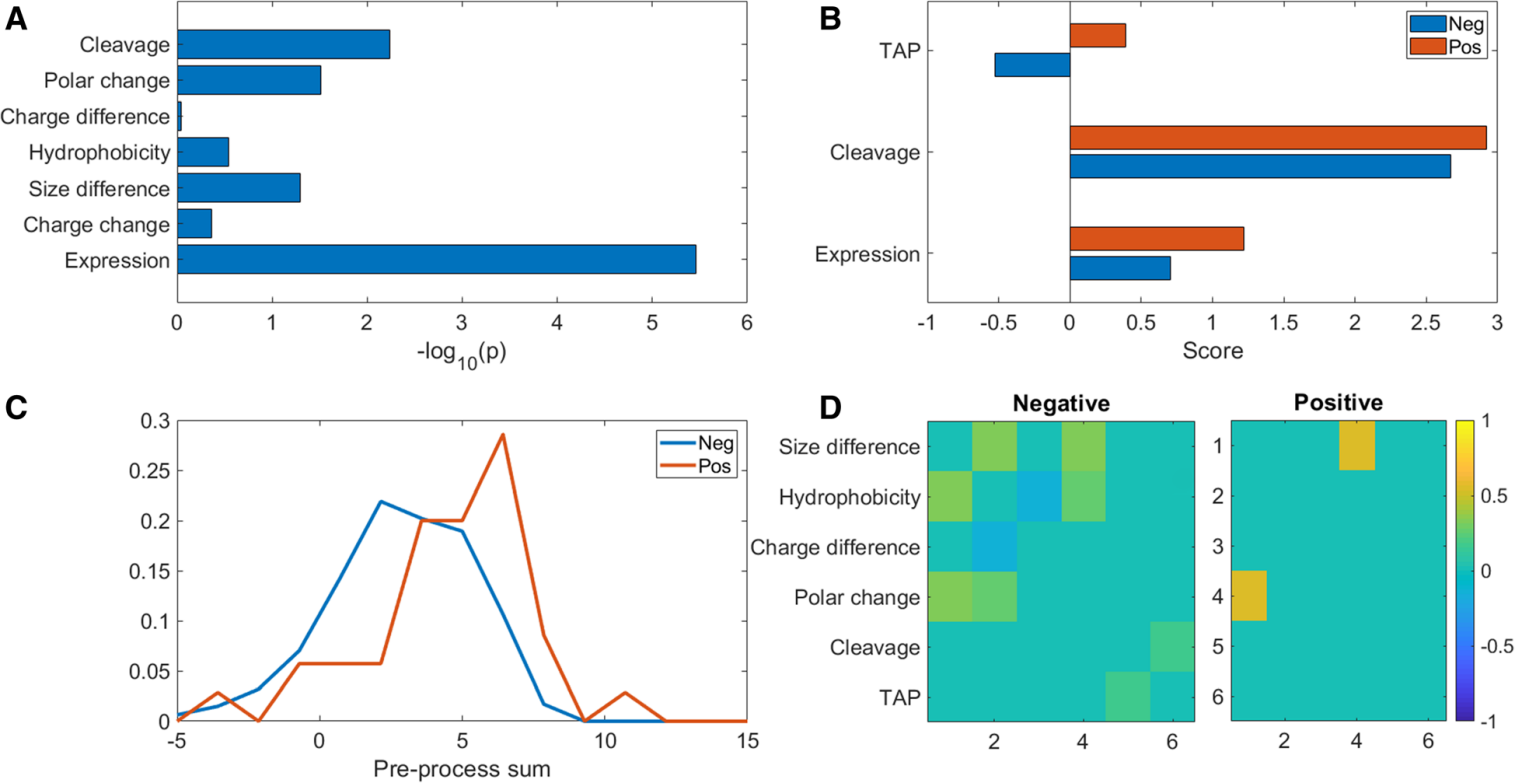
**a**. -log 10 of *p* value for Kolmogorov Smirnov test for similarity between distribution of positive and negative peptides (peptides inducing and not inducing a T cell response). **b**. Average values for positive and negative groups of all measures with significant differences between groups. **c**. Histogram of sum of log expression, TAP binding score and cleavage score. One can clearly see a difference between the groups. **d**. Correlation heatmap of positive and negative groups for all measures. Only correlations with a *p* value below 0.005 were plotted Rows with no significant correlations were removed. The row and columns are the same properties

To test whether the measures above differed between peptide inducing and not inducing a T cell response, we computed the distance of the distributions of each measure in MHC binding peptides activating and not-activating T cells. We used the Kolmogorov Smirnov (KS) distance and found five features significantly different between activating and non-activating peptides (Fig. 2a), mainly expression level, TAP and cleavage scores. Thus, as expected the main element differentiating between epitopes that can and cannot induce a response is the number of peptides reaching the ER. To test that peptides inducing a T cell response have higher RNA expression levels and cleavage and TAP binding probability, the average of the positive and negative groups was computed for all three measures. We observed that the positive (T cell response inducing) peptides have higher averages on all scores (Fig. 2b). To further demonstrate this point, we computed the histogram of the sum of the three scores showing a clear difference between peptides inducing a response (Pos) and peptides not inducing one (Neg) in Fig. 2c.

It is important to note that the MHC binding level was not included in the current analysis, since in all studied peptides (both positive and negative) were predicted to bind MHC. We repeated the analysis for a combined dataset containing the Me. and the epitopes from the Tantigen database of peptides from different tumors, with similar results (data not shown). We focused on MHC binding peptides and thus eliminated peptides that had low score/chance to bind to the specific MHC of this patient. It is worthy to note that including non-MHC binding peptides in the negative set would significantly increase the precision of a resulting classifier (e.g. Kim et al. [12]), but the goal of the current analysis is to add another layer of prediction beyond MHC binding, and thus such peptides were removed.

Beyond the number of peptides reaching the ER, we tested whether the properties of the mutation could affect the T cell response. While there were no significant differences in the average of each studied property, the correlation between these properties (listed in Table 2) differed between the positive and negative groups (i.e., peptides inducing/not inducing a response) - Fig. 2d.

### Machine learning based classifier

**Fig. 3:**
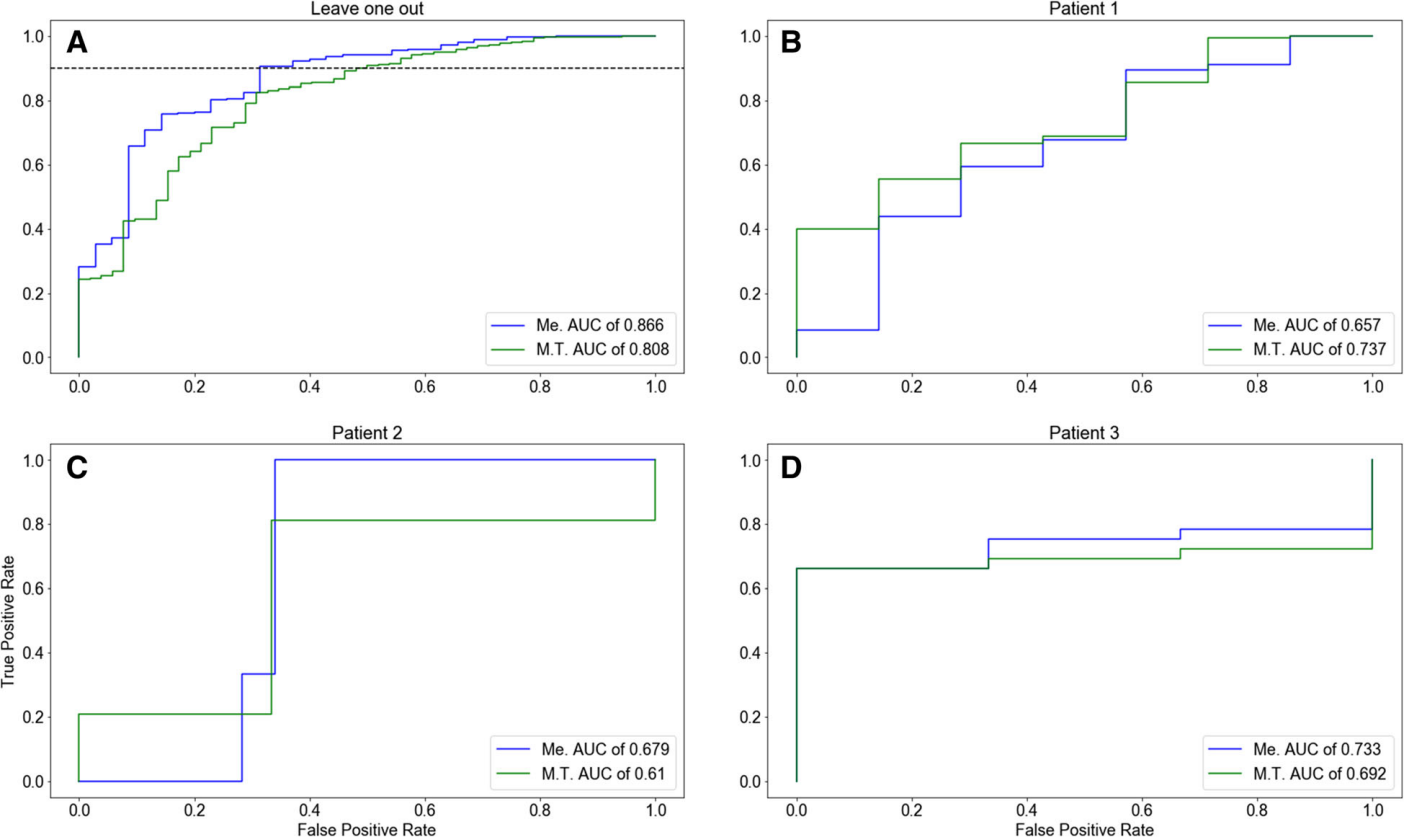
Subplots of ROC curves (**a**) Leave one out test for each one of the datasets. The AUC for the test on melanoma dataset is 0.86. **b**-**d** In the ROC curve for three different patients, the prediction was with the classifiers used to generate the test in (**a**). The horizonal dashed line in (**a**) indicates the threshold of 90% of the data to be true positive

We have used the features above and produced two binary classifiers for the induction of a T cell response by neo-antigens for the Melanoma (Me.) and M.T. datasets. We also utilized the features in Table 2 as the input for a Random Forest classifier, developed with a Leave One Out approach. The resulting AUC was 0.86 (Fig. 3a) (the train AUC was 0.98) for the Me. classifier. For the M.T. classifier, a slightly lower AUC of 0.80 was obtained. However, as we show, when tested on datasets accumulated in a different experimental setup, both perform similarly. In both cases (Me. and M.T.), a 50% TN (True Negatives) and approximately 90% TP (True Positives) can be achieved in both classifiers (dashed line in Fig. 3a); thus, a fast pre-screening stage can be done with no loss of sensitivity. Such a prescreening stage can be highly useful for testing only half the peptide for T cell activation.

**Fig. 4:**
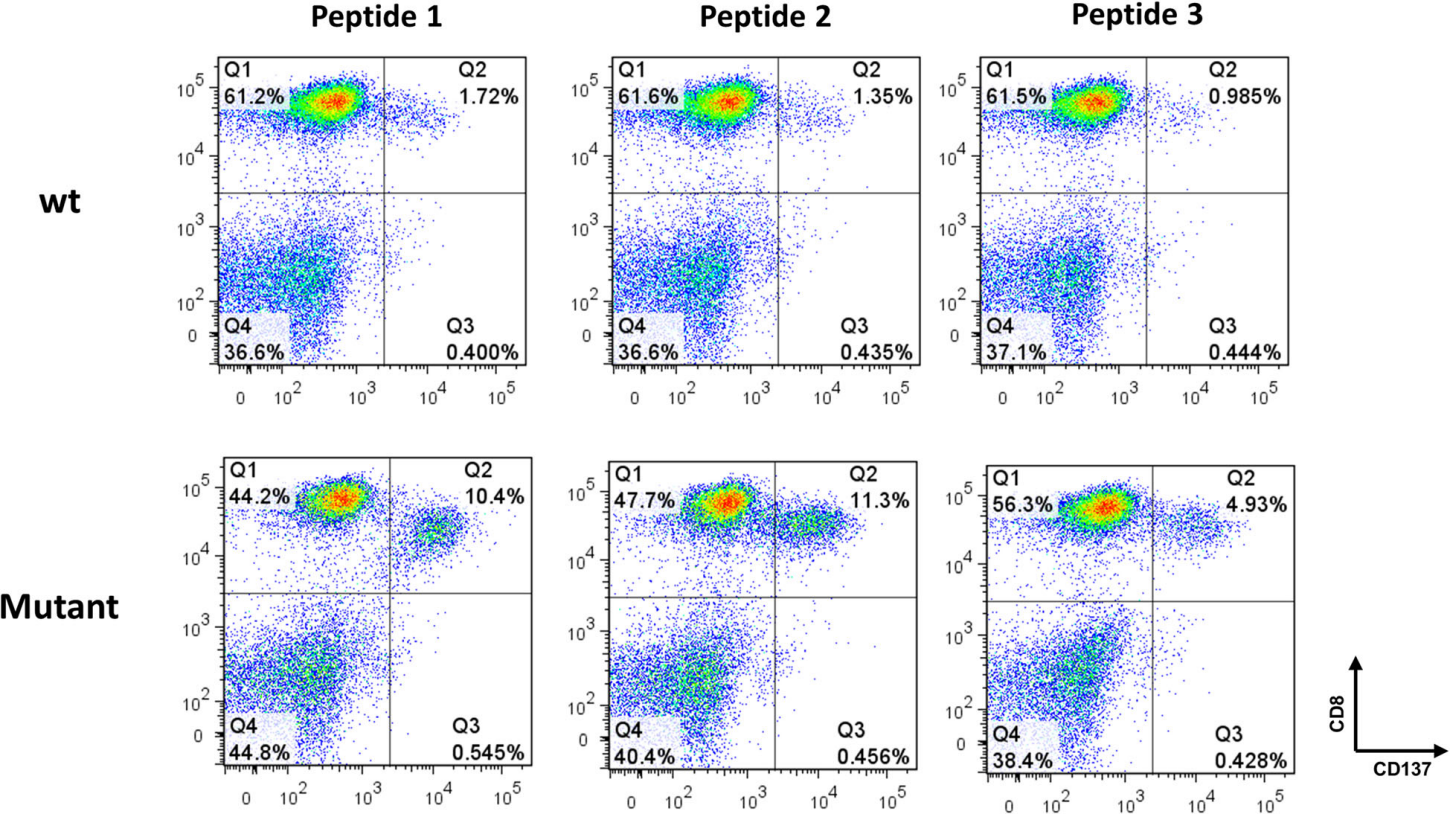
Experimental validation of T cell response. TIL culture of patient 1 recognized 3 neoantigens, but not the corresponding wildtype peptides. Following pulsing with 10 μg/ml of 25-mer mutant or wt peptide overnight, EBV-transformed autologous B cells B-LCL were co-cultured with T-cells from TIL culture from patient 1. 16 h after the beginning of the co-culture, these cells were co-stained for CD137 (41BB) and CD8+ and analyzed by flow cytometry. The double positive population is indicated in quadrant Q2

Finally, to verify our classifier, we screened exome data from 3 additional melanoma patients; the residues surrounding the amino acids resulting from non-synonymous mutations (total length of 25 aa) were screened to identify putative mutated epitopes that could trigger cognate T-cell activation. T cells from these patients were co-cultured overnight with EBV-transformed autologous B cells (B-LCL), pulsed with predicted reactive peptides. Following co-incubation, the upregulation of a T-cell activation marker as a surrogate for T-cell activation, CD137 (41BB) was determined on T cells by flow cytometry [24]. In Fig. 4, we show an example of the staining of TIL cultures derived from patient 1 that were previously incubated with 3 different predicted neo-peptides and their W.T. counterparts. We observed a significant upregulation of CD137 ranging between 4.9–11.3% in T-cells co-cultured with neoepitopes compared to background staining (around 1–2%) seen with W.T. peptides. These results exemplify the existence of T-cells specific for the predicted neoepitopes in TIL cultures.

We examined each patient individually (Fig. 3b-d), and obtained AUC values of 0.73, 0.67 and 0.65 for the three melanoma patients tested (Fig. 3). The origin of the large differences between this experiment and the first two datasets is the completely different experimental setups. The significant AUC in the validation is evidence for the robustness of our prediction to variation in the experimental settings.

To further test the applicability of the method, we computed for the test set in each sample different measures, including the AUC, the accuracy, F1, as well as the fraction of positive samples maintained when 50% of negatives were removed, and similarly the fraction of negative samples remaining when keeping 50% of positives (Table 3). One can clearly see that the vast majority of negative peptides can be removed, maintaining half the positive peptides, and similarly, the vast majority of positive peptides can be maintained, while removing 50% of negative peptides.

**Table 3:** Classifier properties

	ME	M.T.	Pat 1	Pat. 2	Pat. 3
AUC	0.809	0.868	0.657	0.679	0.733
Fraction of Negatives kept when loosing 50% of positives	0.101	0.051	0.322	0.000	0.246
Fraction of positives kept when loosing 50% of negatives	0.865	0.914	0.714	0.660	1.000
Accuracy	0.753	0.810	0.662	0.830	0.831
F1	0.391	0.331	0.207	0.795	0.214

For each dataset we provide, the AUC and F1 value as well as the max accuracy on the test set. Similarly, we provide the fraction of negatives maintained when keeping 50% of negative and the fraction of positives when removing 50% of negatives

## Discussion

CD8+ T-cells are undoubtedly central to the anti-tumor response against cancer whether when considering the impact of immune contexture [25], immune checkpoint or using adoptive transfer of tumor specific T-cells [26–29]. In this context, we and others have shown that a major target for anti-tumor T-cells are neo-epitopes derived from mutated antigens [30–32]. To detect such epitopes, one can employ a strategy encompassing 2 or possibly 3 mains steps:

A) Identification of missense mutations using deep-sequencing,
B) detection of MHC binding peptide appropriate for the host, and sometimes,
C) prediction which of these peptides are properly processed by the Proteasome and bind TAP.

While highly precise algorithms exists for these three stages [2, 3, 33, 34], the resulting predicted MHC binding neo-peptides do not induce a CD8 T cell response in the majority of cases [4, 35, 36]. This can be due to the fact that either T-cells specific for these epitopes never existed to begin with, were eliminated during negative selection, underwent anergy or ceased to exist. Another possibility is that the epitope itself is not potent enough to generate a detectable reactivity.

In the present work, we have shown that it is possible to predict the immunogenicity of neoepitopes based on a fourth layer for this analysis, which is a prediction of the peptide passing the filters above that can induce such a T-cell response, using the properties of the protein and of the mutation. We propose to add this layer to peptide prediction pipelines to improve current methodologies.

A crucial element affecting the response is the expression level of the protein carrying the peptide. We have here shown that such an association is also critical for T cell stimulation/activation by neo-antigens [19]. Another important element is the charge difference of the mutation itself. Such a difference may represent the important effect of charge on T cell-epitope binding [37].

An alternative approach would be to optimize the MHC binding ignoring the other elements. It has been shown that immunogenicity is associated with strong MHC binding [38–40]. However, this approach limits the scope of the possible neo-antigen to excellent binders, and such binders are not always found for all HLA alleles.

While CD8+ T-cells are considered a central driver of the anti-tumor activity of the immune response, recent reports suggest that the adoptive transfer of CD4+ T-cells can lead to tumor cytotoxicity and clinical response, both in animal models and in patient studies [41]. There are currently limited prediction algorithms for MHC class II epitope presentation and pre-processing [42, 43].

In conclusion, we have developed and used herein a novel predictive algorithm that would enable the more precise identification of neo-epitopes that can facilitate CD8+ T-cell activation. We trust that this algorithm will contribute to the design of more precise and potent immunotherapies targeting neo-epitopes.

## Methods

### Datasets studied

Me. dataset: we previously described our experimental screening methodology [7]. Briefly, following exomic sequencing and RNA-Seq analysis of tumor samples collected from eight metastatic melanoma patients, we predicted candidate nine and ten amino acid peptides containing mutated residues derived from proteins with a minimum FPKM of 1 using the IEDB prediction algorithm available. T cells were tested for reactivity to T2 cells pulsed with predicted epitopes in cytokine release assays at an effector to target ratio of 1:1. Epitopes were deemed positives if yielding repetitive (*n* > 3) significant cytokine secretion of at least 3 times above background.

M.T. dataset: we download from the Tantigen database published neoepitope epitopes that were published in previous papers [22]. Those epitopes are from a wide variety of tissues, not limited only for melanoma tissue and not only for a specific MHC class. We combined the first dataset (Me.) and the epitopes from Tantigen to one dataset.

### Prediction of neoantigens

Analysis of whole exome sequencing identified non-synonymous mutations from tumor and matched normal tissue. RNA-seq narrowed down the selection using threshold of expression level. The NetMHCpan predication algorithm was applied to further eliminate peptides by prediction of the Ic50 for to the specific HLA-molecules of the patient.

### Detection of neoantigen specific –T-cells

For each candidate, neoantigen 2 × 10e6 EBV-transformed autologous B cells B-LCL were pulsed with 10 μg/ml of a 25-mer mutant peptide in a 96-well plate and incubated overnight at 37 °C and then washed twice. Co-culture assays were performed by adding 0.5 × 10e5 T cells to each well, followed by an overnight incubation at 37 °C. Reactivity of neo-antigen specific T cells was determined by CD137 expression measured by flow cytometric analysis. WT peptides served as control.

### Statistical methods

We used the Mann Whitney score to compare the median values of each measure in the distributions. To further test for differences in the distribution not apparent in the median, we used the Kolmogorov-Smirnov Distance.

### Learning and evaluation

The TCR binds to the epitope and to the MHC. The vast majority of presented epitopes do not induce a T cell` response. We developed a predictor of T cell activation for MHC binding peptidesbased on the biochemical character of the peptide presented and on the expression level of the antigen. We calculated the difference in the following parameters between the unmutated peptide. and the mutant at the mutation site: Charge, hydrophobicity, size, polarity.

We used a Random Forest algorithm (Matlab) with the following hyper-parameters 5000 estimators, min leaf size (minimum number of observations per tree leaf) 10, cost matrix (penalty) [[0, 0.15], [1, 0]]. To evaluate the performance, we used a leave one out method on the positives (the amount of the positives is much smaller than the negatives). In order to get accurate and unbiased results, we maximized the positives in the train). We had 35 positives samples and 450 negatives samples??that?? we??learned?? 35 times on 34 positives and 435 negatives and validated on the remains. The ROC curve is the score for the all data obtained from the validation method. The negative fraction in the test was 1:12.

The main measure we used to examine the performance is the area under the curve (also known as c-statistics) obtained from the surface under the curve describing the FPR (false positive rate) vs the TPR (true positive rate).
